# Fully Scalable Fuzzy Neural Network for Data Processing

**DOI:** 10.3390/s24165169

**Published:** 2024-08-10

**Authors:** Łukasz Apiecionek

**Affiliations:** Faculty of Computer Science, Kazimierz Wielki University in Bydgoszcz, Jana Karola Chodkiewicza 30, 85-064 Bydgoszcz, Poland; lukasz.apiecionek@ukw.edu.pl; Tel.: +48-606-120-335

**Keywords:** fuzzy logic, artificial neural network, Industry 4.0, data processing

## Abstract

The primary objective of the research presented in this article is to introduce an artificial neural network that demands less computational power than a conventional deep neural network. The development of this ANN was achieved through the application of Ordered Fuzzy Numbers (OFNs). In the context of Industry 4.0, there are numerous applications where this solution could be utilized for data processing. It allows the deployment of Artificial Intelligence at the network edge on small devices, eliminating the need to transfer large amounts of data to a cloud server for analysis. Such networks will be easier to implement in small-scale solutions, like those for the Internet of Things, in the future. This paper presents test results where a real system was monitored, and anomalies were detected and predicted.

## 1. Introduction

Industry 4.0, also known as the Fourth Industrial Revolution, refers to the current trend of automation and data exchange in manufacturing technologies. One of the key features of Industry 4.0 is the use of data analytics and machine learning to optimize manufacturing processes and improve product quality. This involves collecting and analyzing data from sensors and other sources throughout the manufacturing process, and the use of this information to identify inefficiencies, predict maintenance needs, and optimize the production schedules [1]. Another important aspect of Industry 4.0 is the use of interconnected devices and systems, known as the “Internet of Things” (IoT), to create a more integrated and responsive manufacturing ecosystem. This comprises connecting machines, sensors, and other devices to each other and to the cloud, allowing users to monitor and control manufacturing processes. Industry 4.0 is also seen as an innovation driver, enabling manufacturers to develop new products and services more quickly and efficiently than ever before. By using advanced digital technologies to create smarter, more flexible production systems, manufacturers can respond more rapidly to market-changing demands and customer preferences. Overall, Industry 4.0 represents a significant opportunity for manufacturers to improve productivity, increase efficiency, and drive innovation in an increasingly competitive global market. The proposed solution has the following advantages of using it for local data analysis on IoT devices:

- Decentralization of data analysis: IoT devices can independently process data and make decisions, which increases their autonomy and reduces the latency associated with transmitting data to a central server;
- Optimization of energy consumption: thanks to local data processing, IoT devices can more efficiently manage their energy usage, which is crucial for battery-powered devices;
- Increased data security through local processing: processing data on the device reduces the risk of data interception during transmission, therefore enhancing information security;
- System modularity: with local processing capabilities, IoT systems can be more easily expanded with new devices without the need to modify the central infrastructure;
- Shorter response time to events: local analysis and decision-making allow for faster responses to anomalies or other events in real-time;
- Reduced data transmission costs: fewer data to be sent to central servers means lower costs associated with data transmission and less demand for network bandwidth;
- Lower infrastructure cost: reduced requirements for central data centers and a reduced need for complex cloud-based analytical algorithms;
- Local customization of algorithms: IoT devices can be equipped with algorithms tailored to the specific needs of local users or operating conditions;
- Offline functionality: devices can continue their operations and analyze data, even in areas with poor network access, which is crucial in remote or hard-to-reach locations.

The primary value of this paper lies in its proposed solution to an academic challenge. The goal of this article is to present a solution for data analysis on edge devices, where analysis requires the following:

- Data transmission to a central analysis center, which causes data transmission energy costs, and a steady connection may not always be available;
- Alternatively, the need for local data analysis, which, over a long period, with battery-powered devices, will generate energy consumption.

The use of a fuzzy artificial neural network with Ordered Fuzzy Numbers can result in energy savings in some cases due to lower computational power requirements while maintaining the same quality of solution. The proposed solution has been tested in terms of solving logical problems (OR, AND, and XOR functions) and linear function approximation. In the mentioned cases, the proposed solution achieved very good results in solving the same problems, but with smaller network architecture requirements. The author of this article compares the proposed solution with commonly used deep networks and LSTM networks. The author suggests that an artificial neural network can be built using fuzzy logic, particularly employing Ordered Fuzzy Numbers (OFNs) within its neurons. Subsequently, he proposes its application in compact IoT devices, aligning with the principles of Industry 4.0. In this paper, a brief introduction to the fuzzy network with OFN is presented, and some test results are provided. Therefore, I conducted research on my hypothesis, implemented a network, and compared it with an ordinary network that could be used on devices with limited computational power.

## 2. Short Review of Fuzzy Neural Network

Numerous papers explore the realm of fuzzy neural networks. Some researchers attempted to utilize the McCulloch–Pitts model of a neuron, which has been expanded into a more versatile framework allowing neuron activity to be a “fuzzy”, rather than an “all-or-none” process [2]. Others propose architectures of fuzzy neural networks featuring triangular fuzzy weights [3]. These networks are capable of handling both fuzzy and real input vectors, resulting in fuzzy vector outputs. The input–output relationship of each unit within the fuzzy neural network is determined by Zadeh’s extension principle. Many studies utilize fuzzy signals and/or weights [4]. For example, Paulo Vitor et al. introduced a new logical fuzzy neuron, termed null-unineuron, based on the concept of null-uniform, contributing to the architecture of evolving neuro-fuzzy models [5]. The primary application of fuzzy networks is to develop controllers that outperform conventional ones [6,7,8,9,10,11,12,13,14,15,16,17]. In [18], the authors proposed a novel multi-functional recurrent fuzzy neural network. Their architecture comprised two fuzzy neural networks employing Takagi–Sugeno–Kang fuzzy rules; one network is utilized for output generation, while the other determines the system’s state, with a feedback connection between them. Additionally, there have been studies that provide reviews of fuzzy systems and artificial neural networks [19]. In the paper referenced as [20], the author introduced a novel approach to fuzzy logic systems—the adaptive network-based fuzzy interface system (ANFIS). This approach differs from traditional fuzzy logic systems in that it utilizes not only logical rules, but also connections between layers of the network that are not necessarily close in proximity. This allows for a more flexible and adaptive system that can better handle complex and dynamic problems. In [21], the authors proposed a low-rank tensor regularized graph fuzzy learning method for multi-view data processing in which fuzzy learning is adopted to make graph clustering a soft clustering method. The fuzzy learning method is also used in many solutions [3,22,23]. It can be noticed that some hybrid approaches to fuzzy supervised learning were developed [24].

In this study, the author introduces an innovative approach absent from existing literature. We employ a conventional deep neural network and transform individual neurons into fuzzy neurons through the application of Ordered Fuzzy Numbers arithmetic. This adaptation enables us to utilize not only the triangular form of fuzzy numbers, as seen in the ANFIS model, but also the trapezoidal form.

## 3. Fuzzy Neural Network with Ordered Fuzzy Numbers

**Figure 1:**
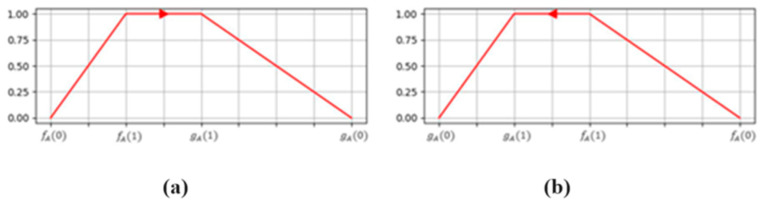
Example of an Ordered Fuzzy Number. (**a**) Positive; (**b**) negative.

Artificial Neural Networks, encompassing Deep Networks, are widely favored tools within the realm of Artificial Intelligence today. These networks are built using neurons, and one common type of neuron is the McCulloch–Pitts neuron. This neuron can have many inputs, each of which is assigned a weight and produces one output. Professor Witold Kosiński, along with his team, proposed a generalization of fuzzy numbers by introducing a trend to them, which proved to be helpful in many event analyses. Ordered Fuzzy Number is an ordered pair $A=\left(f_{A}{,g}_{A}\right)$ of continuous functions $F:f_{A}{,g}_{A}:\left[0,1\right]\to R$ referred to respectively as parts (presented in Figure 1):

- ${up}_{A}$_A—beginning, rising slope;
- ${down}_{A}$—end, falling slope.

Basic arithmetic operations on Ordered Fuzzy Numbers:

- Sum: ordered fuzzy number $C=\left(f_{C}{,g}_{C}\right)$ is the sum of numbers $A=\left(f_{A}{,g}_{A}\right)$ and $B=\left(f_{B}{,g}_{B}\right)$, when:
  (1) $$\forall_{y\in \left[0,1\right]}\left[f_{A}\left(y\right)+f_{B}\left(y\right)=f_{C}\left(y\right)\;\wedge \;g_{A}\left(y\right)+g_{B}\left(y\right)=g_{C}\left(y\right)\right],$$
- Difference: ordered fuzzy number $C=\left(f_{C}{,\;g}_{C}\right)$ is the difference between numbers $A=\left(f_{A}{,\;g}_{A}\right)$ and $B=\left(f_{B}{,\;g}_{B}\right)$, when:
  (2) $$\forall_{y\in \left[0,1\right]}\left[f_{A}\left(y\right)-f_{B}\left(y\right)=f_{C}\left(y\right)\;\wedge \;g_{A}\left(y\right)-g_{B}\left(y\right)=g_{C}\left(y\right)\right],$$
- Multiplication by scalar: ordered fuzzy number $C=\left(f_{C}{,\;g}_{C}\right)$ is the result of the multiplication of number $A=\left(f_{A}{,\;g}_{A}\right)$ by scalar r∈R, when:
  (3) $$\forall_{y\in \left[0,1\right]}\left[{r\cdot f}_{A}\left(y\right)=f_{C}\left(y\right)\;\wedge \;r\cdot g_{A}\left(y\right)=g_{C}\left(y\right)\right],$$
- Product: ordered fuzzy number $C=\left(f_{C}{,g}_{C}\right)$ is the product of numbers $A=\left(f_{A}{,g}_{A}\right)$ and $B=\left(f_{B}{,g}_{B}\right)$, when:
  (4) $$\forall_{y\in \left[0,1\right]}\left[f_{A}\left(y\right)\cdot f_{B}\left(y\right)=f_{C}\left(y\right)\;\wedge \;g_{A}\left(y\right)\cdot g_{B}\left(y\right)=g_{C}\left(y\right)\right],$$
- Quotient: ordered fuzzy number $C=\left(f_{C}{,\;g}_{C}\right)$ is the quotient of numbers $A=\left(f_{A}{,\;g}_{A}\right)$ by $B=\left(f_{B}{,\;g}_{B}\right)$, when:
  (5) $$\forall_{y\in \left[0,1\right]}\left[f_{A}\left(y\right)/f_{B}\left(y\right)=f_{C}\left(y\right)\;\wedge \;g_{A}\left(y\right)/g_{B}\left(y\right)=g_{C}\left(y\right)\right],$$

While fuzzy networks have been explored in the previous literature [21], none have incorporated Ordered Fuzzy Numbers (OFNs) in Artificial Neural Networks. The OFNs were chosen for use in artificial neural networks because:

- They allow calculations to be performed while avoiding the identified drawbacks of traditional L-R numbers;
- No attempt has yet been made to implement a network using this solution, so that is why this is a novel solution.

To build a fuzzy network, a modification of the McCulloch–Pitts neuron model has been suggested. Nonetheless, rather than employing standard numerical values, inputs, weights, and outputs are depicted using OFN notation [22]. OFN notation is used to express imprecision and uncertainty in numerical values. To perform arithmetic operations with OFN numbers, two specific steps are followed:

Step 1: The calculation involves multiplying the input values by their corresponding weights and then adding up the products to obtain a final value:

(6) $$S=W_{0}+\sum_{i=0}^{n}X_{i}W_{i},$$

Step 2: Then, the output using the OFN arithmetic is calculated:

(7) Y=f(S)

The elements within S, W, X, and Y are represented as values in OFN notation. This methodology necessitates subsequent layers to be implemented:

- First layer—a fuzzification process is employed to convert input data into the network into OFN notation;
- Last layer—defuzzification is utilized for processing the output data from the network;
- Deep layer—network learning/training algorithms are adapted to operate effectively with OFN network arithmetic in network layers.

In the proposed network, a deep fuzzy network is employed, where traditional neurons are replaced with fuzzy neurons whose weights are directed fuzzy numbers, and the arithmetic of these numbers is used for calculation. The first layer is responsible for fuzzifying the input data, which is crucial from the perspective of the solution’s application. Each implementation will require a specific approach to the fuzzification process and the representation of data in the domain of fuzzy numbers. Multidimensional data will also require analysis and the introduction of fuzzification methods. However, many solutions have already been developed from which researchers can draw methods for data fuzzification for further analysis. This process should not pose a problem. The subsequent deep layers are responsible for generalizing the information. A deep network with fully connected neurons, a type of FFN, has been developed. The last layer is responsible for defuzzifying the result. In the literature, one can also find numerous methods for defuzzifying results, allowing a return from the domain of fuzzy numbers to the domain of real numbers.

**Figure 2:**
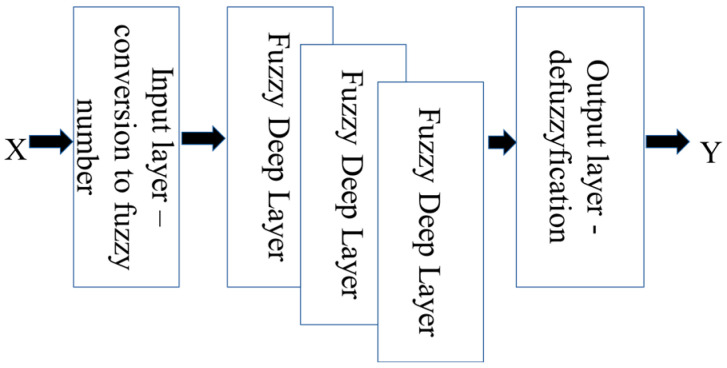
Proposed novel fuzzy network with OFN.

The operational framework of the developed deep network is illustrated in Figure 2.

A network according to these rules with a fuzzy learning algorithm was developed. The test was performed. This network was prepared in Python language.

The code for preparing the network layer is presented in Table 1. The Layer class has three methods, __init__(), forward(), and backward(), which define the behavior of the layer during both forward and backward passes of the network. The __init__() method initializes two instance variables, input and output, which will be used to store the input and output of the layer during the forward pass. The forward() method takes an input and performs some operations on it to produce an output. However, the pass keyword indicates that no specific operations are defined here and it must be overridden by a subclass that inherits from Layer. The backward() method takes the gradient of the loss function concerning the output of the layer and computes the gradient with respect to the input. As with forward(), the pass keyword indicates that this method must also be overridden by a subclass. Overall, this code defines the basic structure for a neural network layer, but specific operations and gradients need to be implemented in a subclass.

The code presented in Table 2 defines a subclass, Dense, of the Layer class. This subclass represents a fully connected neural network layer, where each neuron in the layer is connected to every neuron in the previous layer. The __init__() method takes two arguments: input_size and output_size, which represent the number of neurons in the previous layer and the current layer, respectively. The method initializes the layer’s weights and bias parameters as two NumPy arrays of the specified shapes. The OFN() function represents the Ordered Fuzzy Number class.

The forward() method takes an input tensor, performs the linear transformation by multiplying the input tensor by the weights, and adds the bias. The method stores the input tensor in the self.input variable, which is used in the backward pass to compute gradients. The method then returns the output tensor.

The backward() method takes the gradient of the loss function with respect to the output of the layer, output_gradient, and the learning rate. The method computes the gradient of the loss function concerning the weights, weights_gradient, and updates the weights and bias parameters using the gradient descent optimization algorithm. Finally, the method returns the gradient of the loss function with respect to the input of the layer, which will be used to propagate the gradient backward to the previous layer. Overall, this code defines a fully connected neural network layer with the ability to perform forward and backward passes, and update its parameters during training.

The code presented in Table 3 defines an activation function layer with the ability to perform forward and backward passes and compute the gradient of the loss function with respect to its input. A NumPy table np was used for multiplication calculations.

Also, the mean square error function was defined. The code presented in Table 4 defines an example of a network with two layers:

- First layer with 2 inputs and 4 neurons;
- Second layer with 4 inputs and 2 neurons;
- Second layer with 2 neurons on input side and 1 output.

The activation function used in this network is tanh(). The authors are still performing experiments with different activation functions.

The code in Table 5 represents the implementation of training a neural network using the backpropagation algorithm. There is a loop that runs for a specified number of epochs, using X and Y and calculating the error, and the learning rate parameter is then used to update the weights in each layer during backpropagation.

Finally, the class for OFN definition (Table 6), which could be seen in the previous code, was developed. In this class, the operators used for arithmetic on the OFNs were defined: +, -, *, /. This allows the use of the NumPy library for the calculation of the necessary values during network learning process. This class is used for generating weights and bias in a proposed network, which is presented in Table 2.

One of the important functions in this class is the definition of the defuzzification function.

One of the most important functions of the OFN class is the defuzzification function. A lot depends on the value of this function, as it affects the value that the fuzzy variable will take in the domain of real numbers. Different defuzzification functions will therefore return slightly different results. To date, many defuzzification functions have been developed that can be applied to OFNs, such as:

- LOM—Largest of Maximum is a method used in fuzzy logic to determine the degree of probability for each element of a fuzzy set by finding the maximum membership value. This approach is commonly applied when assigned values carry greater significance, and is particularly useful in decision-making systems where minimizing errors and achieving high-quality defuzzification are crucial. Essentially, LOM selects the largest value from the maximum membership values of the fuzzy set elements, making it a valuable tool for optimizing the accuracy and reliability of fuzzy logic-based systems;
- MOM—Mean of Maximus is a method used in fuzzy logic to determine the degree of probability for each element of a fuzzy set by calculating the arithmetic mean of the maximum membership values. This approach is commonly applied when different membership values are equally important, unlike the LOM method, where certain values hold more significance. MOM is particularly useful in decision-making systems where less precision is required compared with LOM, but where different membership values must still be considered. Essentially, MOM provides a way to balance the importance of different membership values, resulting in a more versatile and flexible approach to fuzzy logic-based systems;
- FOM—First of Maximus is a method used in fuzzy logic to determine the degree of probability for each element of a fuzzy set by selecting the maximum value that occurs first on the variable axis. This approach is commonly applied when the most important value is the degree of membership that first reaches its maximum value, and when a quick decision is required. FOM can be particularly useful in decision-making systems where there is only one degree of membership that is significantly higher than the others, or where precision is not a major concern. Essentially, FOM provides a way to quickly identify the most important value of a fuzzy set, making it a valuable tool for time-sensitive applications;
- Golden Ratio is a method that uses a mathematical constant with a value of approximately 1.618 to determine the real value.

There are, of course, many other defuzzyfication methods and not all of them have indications for use based on their characteristic features. Therefore, the selection of the sharpening function will be the subject of further experimental work.

## 4. Monitoring System—Solution Tests

A monitoring system that used Java on web browsers was used. During the test, the data analysis was performed. As part of the task, the system of logs used in the produced applications was analyzed. Several systems were selected, from which logs were taken for testing, as follows:

- Cafeteria system—application (event) log;
- System of internal legal acts—application (event) log;
- Logs of Apache Tomcat application servers;
- MariaDB database system logs;
- operating system status logs.

As part of the prepared set of logs, they were segregated and segmented, creating a coherent set of logs. A fixed number was assigned to each of the logs, specifying the maximum number of log types per 10,000 for testing. The log number–log type is a dictionary, while the file for training the neural network collects the number of log occurrences in a fixed period of observation time—5 min. This time was chosen so that it was neither too large nor too small and allowed for subsequent summation to periods of, for example, 15 min, 30 min, or 1 h.

As part of the collection of logs, a collection of over 1 million system logs was built, which were classified in accordance with the established type base. This set was used for research on a fuzzy neural network.

In addition, a detailed description of selected anomalies for prediction has been defined, as follows:

- Type 1: Increased number of repetitive system errors;
- Type 2: Specific repetitive sequences of events leading to system failure;
- Type 3: Resource-consuming system actions leading to errors occurring in a short time;
- Type 4: Abnormal events related to an attempt to access the system;
- Type 5: Analysis of trends related to specific actions in the system and finding situations deviating from the norms;
- Type 6: Detecting errors related to communication between different systems;
- Type 7: Detection of point increased load on the system that may suggest errors or attacks.

These data were selected for the following reasons:

- It came from an existing system, thus addressing a practical problem of anomaly detection and prediction;
- It can represent logs from any device, such as a network edge device: router, firewall, access device, or edge sensor solution.

This demonstrates that they can represent an IoT solution, allowing the connection of virtually all types of sensors to the network.

### 4.1. Research Methodology

To present the solution, it was decided to evaluate the developed fuzzy neural network alongside two example solutions: a deep network and an LSTM network. A series of studies were conducted to determine the network size whose parameters would be satisfactory in terms of the results achieved. The research began with the fuzzy network, working on the number of layers and neurons to achieve a practical detection and prediction parameter for anomalies in the developed dataset at around 90%, with the dataset split in an 80/20% ratio (training data, test data). During the studies, different values for the learning rate parameter and varying numbers of epochs were used. It was not assumed that the network had to learn within a specific number of epochs; instead, the focus was on the duration of the network training process, as detailed in the subsequent sections. After establishing a network architecture that yielded satisfactory results, the performances of the traditional network and the LSTM network were evaluated. For the deep network, attention was also given to the training time, and an architecture was sought that would achieve comparable results with the fuzzy network on the test set. Only by increasing the number of layers were comparable results obtained. However, the LSTM network did not achieve results sufficient for anomaly prediction, and the training time did not allow for further experiments, leading to the cessation of its optimization process. This does not imply that a different architecture might not yield better results for this network.

To train the network, the prepared data were labeled with the types and times of anomaly occurrences. These prepared data were then used in the network training process. Subsequently, the data were labeled with the time of anomaly prediction, assumed to be 60 min before the anomaly’s occurrence. Thus, the task of the network was to learn to predict the occurrence of anomalies before they actually happened. This was intended to give the system administrator the necessary time to take action. Next, according to the data division, the network’s performance was tested on the test data, with the assumption that the false positive and false negative rates should be below 5% of the anomaly occurrences. As the end-user was interested in the percentage of correctly detected and predicted anomalies while ensuring a low false positive and false negative rate, the collected results were presented as percentages.

### 4.2. Research Results Achieved

A Fuzzy Neural Network was used, which consisted of the following layers:

- Input layer: 10,000;
- Deep layer: 64;
- Deep layer: 64;
- Output layer: 1.

There were 150 epochs used, which lasted for 15 min on the machine with Intel Core i7 with 8 GB RAM. At first, the anomaly was detected on the data; then, there was a learning process of the network to predict the anomaly one hour earlier than it will occur. The results are presented in Table 7.

According to the type of data, the literature claims that the LSTM (Long–Short-Term Memory) network should be used. LSTM is a recurrent neural network (RNN) structure that has gained popularity in various natural language processing tasks, such as speech recognition, machine translation, and sentiment analysis. Traditional RNNs often face the problem of vanishing and exploding gradients, where the gradients become very small or very large, making it difficult for the network to learn long-term dependencies.

LSTM addresses this problem by incorporating a memory cell that can selectively add, delete, or modify information from previous time steps. This mechanism enables LSTM to preserve long-term dependencies while also mitigating the issue of vanishing and exploding gradients. LSTM networks are trained using a variant of backpropagation called backpropagation through time (BPTT), which optimizes the weights and biases of the gates and memory cell by minimizing a loss function. Common loss functions include the mean squared error or cross-entropy loss.

First, it should be noticed that the learning process of this network lasted much longer. The fuzzy neural network requires only 15 min, while on the same machine, the LSTM network requires approximately 20 min per epoch, so 150 epochs last 50 h. Then, according to the data used, it do not recognize the anomaly or predict them. This network was prepared in Tensorflow opensource library. The LSTM network has the following structure:

- Input layer: 10,000;
- LSTM layer: 64;
- Deep layer 64;
- Output layer: 1.

Then, an ordinal deep neural network was used with three hidden layers. There were more network layers to achieve the results that could be compared with the fuzzy neural network. The following layers were included:

- Input layer: 10,000;
- Deep layer: 512;
- Deep layer: 256;
- Deep layer 64;
- Output layer: 1.

The learning process lasts for approximately 80 s per epoch, so the 150 epochs last for approximately 3 h 20 min. The results are presented in Table 8.

These three presented networks possess the following numbers of neurons in their structure:

- Fuzzy Neural Network with OFN: 129;
- LSTM network: 129;
- Deep Neural Network: 833.

## 5. Discussion

Employing a fuzzy neuron network with fuzzy neurons, utilizing OFN, facilitated the detection and prediction of anomalies. The test results have proven that such a network is effective and comparable to an ordinary network, but requires a smaller number of neurons. Additionally, the learning process is faster than that of a normal deep network. The learning processes for different types of networks were as follows:

- Fuzzy Neural Network with OFN: 15 min;
- LSTM network: 50 h;
- Deep Neural Network: 3 h 20 min.

Of course, the proposed network model has been validated only for logical functions (OR, AND, and XOR), linear function approximation, and the example of anomaly detection and prediction provided, which accounts for an additional 14 applications. In total, this amounts to 18 different use cases for the network with various input scenarios. It does not, of course, cover all current use cases for deep networks or LSTM networks. Comparing across all possible data types is a very time-consuming endeavor. However, the focus of the research was to implement the solution and conduct an initial verification to determine whether it has practical applicability, i.e., whether it can generalize knowledge, perform detection, and make predictions based on prepared data. As the research results show, this is a tool that can be further investigated and may prove to be better in some cases. Certainly, there will be instances where the proposed fuzzy network will not outperform many other solutions, but it can be considered as an additional tool for solving problems. For the fuzzy network, ablation studies were conducted regarding the number of layers and neurons in the network, examining models three to seven layers deep, with the following numbers of neurons per layer: 48, 64, 92, 128, 256, and 512. The studies showed that smaller networks than the target network exhibited an increase in the number of false positives, while a larger number of layers generated fewer true positives. Sensitivity analysis of the developed fuzzy network was mainly conducted during the preliminary training process for linear function approximation. The prepared solution currently has two parameters defining the learning process: learning rate and number of epochs. The learning rate parameter was set to three values: 0.1, 0.01, and 0.001. The best learning results, measured by accuracy, were obtained with a value of 0.01. It was noted, however, that the network required a large number of epochs for function approximation. Nevertheless, training the network for 100,000 epochs took under 2 min on the same computer used for anomaly detection and prediction. As the fuzzy network operated very quickly, the training process was much shorter compared with the deep network implemented via the Keras API in the TensorFlow environment—there was no justification for conducting further studies with the current data (over 90,000 records). However, such studies should certainly be conducted in the future.

There are numerous applications for the proposed solution, especially in compact IoT devices with constrained computational capabilities. It could also be utilized in small companies in their security operational centers for detecting and predicting anomalies in the future.

Using the proposed solution in IoT devices allows avoiding such problems as:

- The necessity of transmitting the data to remote destinations within the area the solution covers;
- The necessity of developing data transmission protocols required for efficiently transferring the data,
- Creating a huge data center for collecting and processing the data—producers do not need to build a cloud architecture data center for collecting and store big amounts of data, which we need to share with other systems;
- Developing the algorithms for big data analysis—the data could be analyzed in small IoT devices and only the results could be passed to the management center;
- The necessity of connecting various devices working with different communications protocols, which could be incompatible—there is no need to build some gateways or converters for them.

To summarize, connecting all the IoT devices to the network will require transferring a lot of data to the servers working in some cloud. This will also generate a lot of traffic in the network. The proposed method provides the possibility to analyze the data in the network border and allows avoiding this unnecessary traffic.

## 6. Limitations

The currently developed solution, of course, has its limitations:

- First and foremost, the solution has not yet been tested with different neuron activation methods, which will be necessary for its widespread application;
- The second limitation of the solution is the necessity of fuzzifying the data. While there are many examples of applying fuzzy logic in the literature, each use of the proposed fuzzy neural network will require a separate analysis of the data’s nature. Based on the nature of the data, it will be necessary to select a fuzzification method or even develop a new one. Developing new fuzzification methods can be a challenging task. Although various defuzzification methods are well-known in the literature, the fuzzification process still requires a lot of work. Of course, there are certain types of data for which fuzzy logic is ideally suited, particularly data defined by intervals;
- The third limitation of the solution is the necessity for possessing extensive expert knowledge encompassing fuzzification, defuzzification, and neural network construction. A specialist using the proposed solution must combine knowledge of fuzzy logic with neural networks, and the selection of fuzzification and defuzzification methods can significantly impact the achieved results. As the use of the solution increases, it is valuable to build knowledge on the selection of these fuzzification and defuzzification methods

Future research also requires examining:

- The possibilities and results achieved with different sharpening functions, considering that there are currently many sharpening methods, and new ones are continually being developed. Studies show that they affect the output result, and their application requires thorough research. It is estimated that they may even impact the network training process, including its speed,
- The impact of random weight selection, where, in the randomization process, weights can take various shapes, not only trapezoidal. Limiting the shape of random weights or their normalization can also affect the network training process, especially the speed of learning and the quality of the obtained results.

The author is aware of the vast amount of research that can and should still be conducted and encourages readers to pursue their own studies.

## 7. Conclusions

This article introduces the concept of employing the OFN arithmetic for IoT solutions tailored to the Industry 4.0 paradigm. The primary innovation of this paper is a novel fuzzy neural network incorporating Ordered Fuzzy Numbers in its weights. As it was proven, such a network could use a lower number of neurons to detect or predict the same information in the data. Also, the smaller structure allows for speeding up the learning process. Therefore, the author claims that this network could be used in the future in reinforcement learning solutions. The author is currently in the process of developing a framework for TensorFlow, an open-source platform. This framework will enable the utilization of various network types, including Convolutional Neural Networks (CNNs) or Long–Short-Term Memory (LSTM) networks.

The proposed solution of Neural Network with Ordered Fuzzy Logic provides improvements in:

- Energy consumption, because it uses fewer neurons in the network architecture than a Deep Neural Network, which provides the same level of accuracy. This was tested during this work and in some previous tests in which the Iris database was used [22]. As a result, it requires less computational power from the processor, thus allowing for the conservation of the energy needed to perform the calculations;
- Data security, because it allowed for the development of a solution for detecting and predicting anomalies in running software. This enables the administrator to make timely decisions and avoid system issues, thereby eliminating software vulnerabilities. Consequently, this leads to an increase in the security of the processed data;
- Reduced data transmission costs, as the solution can be applied at the network edge and allows for data analysis at the point of origin. This avoids the need to transmit data to a data center, thereby eliminating the associated data transmission costs, including the energy required for such transmission.

Of course, further work should involve investigating the proposed network in terms of the selection of defuzzification methods. It is anticipated that different methods will affect the speed of learning and the quality of the achieved results. Similarly, the choice of neuron activation functions will also be the subject of further research.

## Figures and Tables

**Table 1:** The code for the network class layers.

Class Layer
class Layer: def __init__(self): self.input = None self.output = None def forward(self, input): pass def backward(self, output_gradient): pass

**Table 2:** The code for the network’s Dense layers.

Class Dense
class Dense(Layer): def __init__(self, input_size, output_size): self.weights = np.ndarray(shape=(output_size,input_size),dtype=object) self.weights.fill(OFN()) self.bias = np.ndarray(shape=(output_size, 1), dtype=object) self.bias.fill(OFN()) def forward(self, input): self.input = input return np.dot(self.weights, self.input) + self.bias def backward(self, output_gradient, learning_rate): weights_gradient = np.dot(output_gradient, self.input.T) self.weights -= weights_gradient * learning_rate self.bias -= output_gradient * learning_rate return np.dot(self.weights.T, output_gradient)

**Table 3:** The code for the neurons’ activation class.

Class Activation
Class Activation(Layer): def __init__(self, activation, activation_prime): self.activation = activation self.activation_prime = activation_prime def forward(self, input): self.input = input return self.activation(self.input) def backward(self, output_gradient, learning_rate): return np.multiply(output_gradient, self.activation_prime(self.input))

**Table 4:** The code for network definition.

Network Definition
network = [ Dense(2, 4), Tanh(), Dense(4, 2), Tanh(), Dense(2, 1), Tanh() ]

**Table 5:** The code for network training.

Network Training
# train for e in range(epochs): error = 0 for x, y in zip(X, Y): # forward output = x for layer in network: output = layer.forward(output) # error error += mse(y, output) # backward grad = mse_prime(y, output) for layer in reversed(network): grad = layer.backward(grad, learning_rate) error /= len(X)

**Table 6:** The code for class OFN definition.

Class OFN
Class OFN: def __init__(self, x1 = None, x2 = None, x3 = None, x4 = None): self.x1 = random.random() if (x1 is None) else x1 self.x2 = random.random() if (x2 is None) else x2 self.x3 = random.random() if (x3 is None) else x3 self.x4 = random.random() if (x4 is None) else x4 def __add__(self, other): if isinstance(other, OFN): return OFN(self.x1 + other.x1, self.x2 + other.x2, self.x3 + other.x3, self.x4 + other.x4) else: return OFN(self.x1 + float(other), self.x2 + float(other), self.x3 + float(other), self.x4 + float(other)) def __sub__(self, other): if isinstance(other, OFN): return OFN(self.x1 - other.x1, self.x2 - other.x2, self.x3 - other.x3, self.x4 - other.x4) else: return OFN(self.x1 - float(other), self.x2 - float(other), self.x3 - float(other), self.x4 - float(other)) def __mul__(self, other): if isinstance(other, OFN): return OFN(self.x1 * other.x1, self.x2 * other.x2, self.x3 * other.x3, self.x4 * other.x4) else: return OFN(self.x1 * float(other), self.x2 * float(other), self.x3 * float(other), self.x4 * float(other)) def __div__(self, other): if isinstance(other, OFN): return OFN(self.x1 / other.x1, self.x2 / other.x2, self.x3 / other.x3, self.x4 / other.x4) else: return OFN(self.x1 / float(other), self.x2 / float(other), self.x3 / float(other), self.x4 / float(other))

**Table 7:** Test results on fuzzy neural network with Ordered Fuzzy Numbers.

Type of Anomaly	Percentage of Anomaly Detection on Test Data	Percentage of Anomaly Prediction on Test Data
1	95	91
2	94	89
3	94	91
4	91	85
5	92	84
6	93	88
7	92	87

**Table 8:** Test results of deep neural network.

Type of Anomaly	Percentage of Anomaly Detection on Test Data	Percentage of Anomaly Prediction on Test Data
1	96	92
2	92	86
3	91	88
4	90	86
5	91	83
6	91	86
7	94	88

## Data Availability

The data are not publicly available due to privacy restrictions.
